# Survival probabilities and trends for lip, oral cavity and oropharynx cancers in Northern Portugal in the period 2000–2009

**DOI:** 10.3332/ecancer.2018.855

**Published:** 2018-07-31

**Authors:** Luís Silva Monteiro, Luís Antunes, Lúcio Lara Santos, Maria José Bento, Saman Warnakulasuriya

**Affiliations:** 1 Department of Medicine and Oral Surgery, Cancer Research Group, IINFACTS, Instituto Universitário de Ciências da Saúde Norte-CESPU, Paredes 4585-116, Portugal; 2 Cancer Epidemiology Group, IPO Porto Research Center (CI-IPOP), Portuguese Oncology Institute of Porto (IPO Porto), Porto 4200-072, Portugal; 3 Experimental Pathology and Therapeutics Group, IPO Porto Research Center (CI-IPOP), Portuguese Oncology Institute of Porto, Porto 4200-072, Portugal; 4 Department of Surgical Oncology, Portuguese Oncology Institute of Porto, Porto 4200-072 Portugal; 5 King’s College London and WHO Collaborating Centre for Oral Cancer, London SE5 9RW, UK

**Keywords:** oral cancer, lip cancer, oropharyngeal cancer, net survival, Portugal, trends

## Abstract

**Background:**

Oral cancer represents a serious public health problem worldwide. Our aim was to analyse the survival probabilities and trends of patients presenting with lip, oral cavity and oropharynx cancers, who were residents in the north of Portugal.

**Methods:**

Using cancer-registry data, we conducted a population-based study of lip, oral cavity and oropharynx cancers diagnosed in the period 2000–2009, among residents in the north of Portugal. Net survival was estimated using the Pohar-Perme estimator. Excess hazard ratios (for gender, age group, tumour location, stage, residence area and period of diagnosis) were estimated using flexible parametric models.

**Results:**

A total of 2,947 cases (79.5% males) were included of which 18.5% were located on the lip, 56.2% in the oral cavity and 25.3% in the oropharynx. A large proportion of patients were diagnosed in stages III and IV (18.6% and 48.7%, respectively). The 5-year net survival (5yr-NS) for all three cancer sites together was 46% (95%CI 44–48), being 88% (95%CI 83–94), 41% (95%CI 38–43) and 27% (95%CI 23–30) for lip, oral cavity and oropharynx cancer, respectively. The 5yr-NS stratified by tumour stage was 84% (95%CI 78–90) for stage I, 69% (95%CI 63–76) for stage II, 42% (95%CI 37–47) for stage III and 19% (95%CI 16–21) for stage IV. When comparing the periods 2000–4 and 2005–9, no overall improvements in survival were observed. However, when analysed by stage, a significant reduction in the adjusted excess mortality was observed for stages II (*p* = 0.021) and III (*p* < 0.001).

**Conclusion:**

More than half of the oral cavity and oropharynx cancers were diagnosed in advanced stages of the disease, having a low survival probability. Improvements in survival in the first decade of this century were limited to stages II and III, which were the result of changes in hospital cancer care practices.

## Background

Lip, oral cavity and pharynx cancers were in 2012 the sixth most common cancers in Europe for men [Age-standardised rate (ASR): 18.2/100,000] while for women they ranked in the 16th place (ASR: 4.9/100,000) (EUCAN) [1]. A total of 43,704 deaths due to this disease were estimated to have occurred during 2012 in Europe being almost 80% in men [1]. The incidence and mortality rates of lip, oral cavity and pharynx cancers vary widely between European countries and regions of Europe [2, 3]. Hungary was the country with the highest incidence rate (ASR: 23.3/100,000) contrasting with other countries such as Cyprus with a low incidence rate (ASR: 2.7/100,000 habitants) for these tumours [1]. Contrary to other southern European countries, Portugal presents a relatively high incidence rate of these tumours ranking in fourth place in Europe (considering both sexes) and second place in Southern Europe. A total of 2,082 new cases were estimated for the year 2012 (ASR: 15.4/100,000) [1]. Also, an increasing trend in oral cancer was reported since the beginning of the new century in Portugal [2]. This trend was observed in both sexes but it was more evident in females [2]. In terms of mortality, Portugal ranks better (13th) with an estimated mortality rate of 5.5/100,000 [1]. Nevertheless, more than half of the cases in our country are diagnosed in advanced stages, in particular in the north of Portugal and over two-thirds have regional metastasis at diagnosis [4, 5] contributing to low survival from this type of tumours.

The most common histological type of oral cancer is squamous cell carcinoma and [2] tobacco and alcohol consumption constitute the major risk factors for oral cavity cancers and exposure to sunlight for lip cancers [6]. Additionally, human papilloma virus (HPV) infection has been recognised as an important cause, especially for oropharyngeal cancers [6].

In the first decade of this century, there have been some changes in the clinical practice in the northern region of Portugal regarding head and neck tumours according to European Society for Medical Oncology recommendations [7]. Population-based cancer survival analysis is the best way to evaluate the impact of these changes on outcomes. The aim of the present study was thus to analyse the patterns in net survival from lip, oral cavity and oropharyngeal cancers using a population-based dataset of cancer patients diagnosed in the north of Portugal during the period 2000–2009, in order to understand the impact of these changes.

## Methods

Information was extracted from the population-based North Region Cancer Registry of Portugal (RORENO). We considered eligible for analysis all new lip, oral cavity and oropharynx malignant epithelial neoplasms, diagnosed between 1 January 2000 and 31 December 2009, occurring in men and women aged 15 or more and resident in the north of Portugal (including the districts of Braga, Bragança, Porto, Viana do Castelo and Vila Real). We divided the study into two 5 year periods (2000–4 and 2005–9) in order to evaluate possible improvements in survival during this decade. We reclassified all cases according to the 10th revision of the International Classification of Diseases [8]. For the analysis, tumour locations were categorised into three groups: lip (C00), oral cavity (tongue: C01–02, gum: C03, floor of the mouth: C04 and palate and other unspecified parts of the mouth: C05–06) and oropharynx (tonsil: C09, oropharynx: C10 and other ill-defined sites: C14). Malignant neoplasms of major salivary glands (C07–08), nasopharynx and hypopharynx (C11–13) were not considered in this analysis. Tumour stage was reclassified according to the 7th edition of the classification of malignant tumours of American Joint Committee on Cancer [9]. The follow-up of the vital status of each patient was updated until 31 July 2015, using the National Health Service database.

The area of residence (parish) at diagnosis for each patient was classified as rural, moderately urban or urban according to the classification defined by the Statistics Office of Portugal [10].

The association between categorical variables was evaluated using the Chi-square test or Fisher test whenever applicable. Survival time was defined as the time between tumour diagnosis and patient’s death from any cause, end of the study period or date lost to follow-up, whichever occurred first. Net survival from cancer was estimated using the Pohar-Perme net survival estimator [11]. Net survival is a hypothetical quantity that represents the probability of survival in the absence of other causes of death. For the population background mortality, we used the life tables built in the framework of the CONCORD-2 study (http://csg.lshtm.ac.uk/tools-analysis/life-tables/) stratified by sex, age and calendar year. Excess hazard ratios (EHRs) were computed using flexible parametric models [12]. Univariable models were used to assess the prognosis value of each studied variable. A multivariable model was built with all the variables analysed (age group, sex, topography, stage of disease at diagnosis, a period of diagnosis and typology of residence area) but keeping only the significant variables in the final model. Furthermore, to evaluate improvements in survival by the stage of disease, we built different models for each level of this variable. Since we had less than 30% of cases with missing information on stage, we used multiple imputation techniques in the analysis. Variables in the imputation model included all of the variables considered in the substantive model referred above plus vital status, survival time and the Nelson–Aalen estimate of the cumulative hazard as proposed by Falcaro et al [13]. EHRs were estimated using Rubin rules. All calculations were performed using STATA software. Results were considered statistically significant for *p*-value < 0.05.

## Results

A total of 2,947 cases with lip, oral cavity or oropharynx cancer were reported in the study period. The characteristics of the cohort of patients analysed are presented in Table 1. The male-to-female ratio was 3.9:1. The location most commonly affected was the tongue (823; 27.9%), followed by tonsils and oropharynx (630; 21.4%), lip (544; 18.5%), buccal mucosa (311; 10.6%), floor of the mouth (237; 8.0%), palate (160; 5.4%), gums (125; 4.2%) and pharynx (117; 4%). Squamous cell carcinomas corresponded to 92.5% (*n* = 2,752) of all tumours.

For oral cavity and oropharynx cancers, more than half of the patients were diagnosed in stage IV (51.5% and 64.1%), while for lip cancer the most frequent was stage I (69.6%).

Most of the patients were resident in urban areas (71.3%). The proportion of lip cancer patients coming from rural or medium urban areas was significantly higher than this proportion for the two other cancer types (*P* < 0.001).

**Figure 1.**
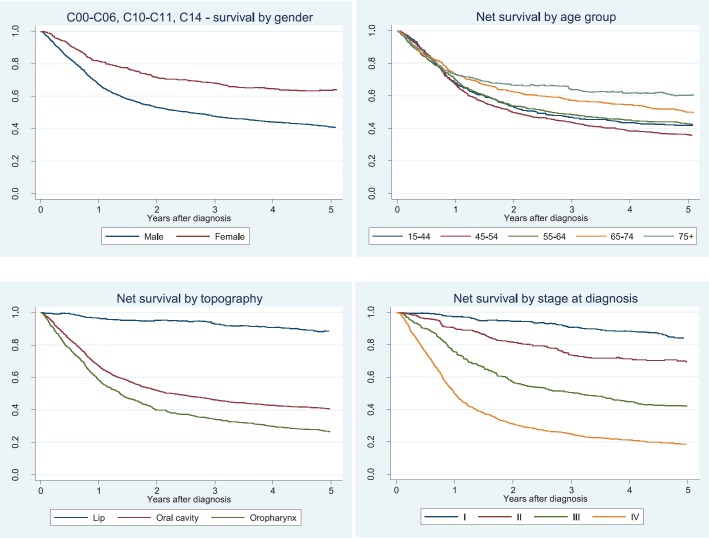
Net survival by gender, age location and clinical stage.

Five-year net survival (5yr-NS) for all cases is presented in Table 2 (and Figure 1). For all cancers, the 5yr-NS probability was 45.8% (95%CI 43.7–47.8). Women presented a better survival than men for all cancer sites. Net survival did not vary by age group, although a slightly better 5yr-NS was observed for the older patients for oral cavity and oropharynx cancers. As expected, the 5yr-NS decreased with increasing stage, ranging from 84% (stage I) to 19% (stage IV) when considering all tumours. No major differences in survival were observed by the typology of residence, although patients living in rural areas had a slightly better survival.

Lip tumours had the best prognosis, followed by oral cavity cancer and then oropharynx (Table 2, Figure 1).

**Figure 2.**
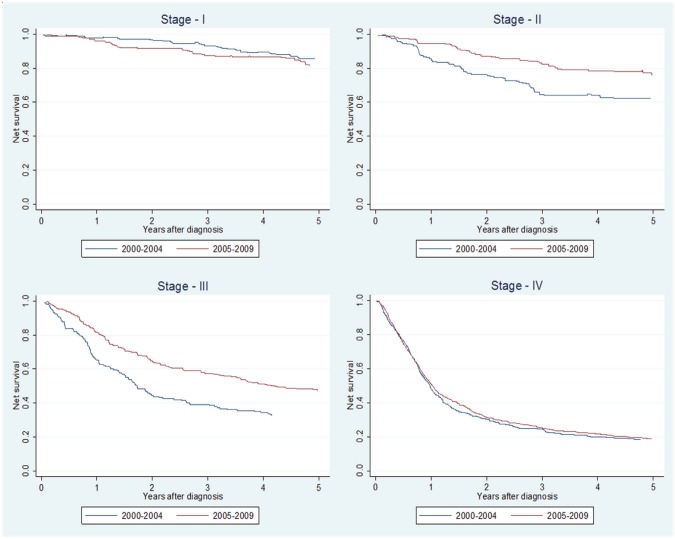
Net survival for the periods 2000–2004 and 2005–2009 analysed by clinical stage (AJCC 7th ed).

Considering all sites together, we observed that the 5yr-NS did not change between the two time periods (45.6% versus 45.8%). Stratifying by tumour site, we observed a slight increase in 5yr-NS in the later period for each of three locations considered (Table 3). We observed an increase in 5yr-NS only in stages II and III (Figure 2).

Female patients had a decreased adjusted excess hazard (EHR = 0.76; 95%CI: 0.63–0.91) and only patients with 65 or plus years of age had an increased excess mortality (65–74: EHR = 1.30, 95%CI: 1.04–1.63 and 75+: EHR = 1.34, 95%CI: 1.02–1.76). Patients with oropharyngeal cancers had an increased excess hazard relative to oral cavity cancer patients (EHR = 1.20; 95%CI: 1.06–1.36) while lip cancers had almost 70% less excess hazard (EHR = 0.32; 95%CI: 0.20–0.52). Stages II–IV presented an increased excess mortality when compared to stage I tumours (Table 4). The patients diagnosed in the most recent period (2006–9) presented a decreased excess mortality (16%) compared to patients diagnosed in 2000–4. A significant reduction in excess mortality (40%) was observed for stages II and III but not for stages I and IV (Table 5).

## Discussion

In this population-based study, we aimed to analyse the survival probabilities and trends of patients with lip, oral cavity and oropharynx cancers among residents in the north of Portugal in the first decade of this century. A low survival probability was confirmed for oral cavity and oropharynx tumours, contrasting with lip cancers that presented a higher survival. For oral cavity and oropharynx cancers, our results are lower than the survival figures observed in many world regions, in particular the European region where survival rates for oral cavity are between 45% and 50% and oropharynx cancers close to 41% [3, 14] while in our region it was 41% and 27%, respectively. Surveillance, Epidemiology, and End Results (SEER) data for the United States in 2009 revealed a 5yrs-NS of 67.2% for oral cavity and pharynx cancers [15].

Lip cancer presented a high survival rate when compared to the other sites studied, similarly to what has been observed in other studies [14, 16, 17].

Significant reductions of excess mortality in the most recent period for oral cavity and oropharynx tumours diagnosed in stage II or III were observed. These observed improvements occurred in the period in which a coordinated multidisciplinary treatment approach, the creation of a surgical and pathological dedicated team, took place in most of the hospitals in the North region of Portugal. We also followed a clear definition of neck dissection (selective or modified radical) depending on the clinical stage and tumour thickness. More effective chemotherapy adjuvant protocols (cisplatin, 5-Fu and taxanes) combined with high-quality radiotherapy were applied. This fact reduced the risk of side effects that associated with nutritional support allowed a better adherence to the treatment protocols. High-quality follow-up tailored by the risk of cancer recurrence was also implemented in this period [18].

However, no survival improvements were observed for early diagnosed tumours (stage I). Probably this fact stems from the treatment performed being standardised and additional improvements being difficult to achieve.

No survival improvements were observed for advanced tumours (stage IV) either. This could reflect the fact that patients with advanced disease have mainly no capacity for supporting systemic treatment including the new types of treatments and new chemo-radiotherapy approaches [19]. It is expected that with the introduction of targeted cancer therapies, immunological oncology approaches for head and neck cancer therapy and photodynamic therapy, survival from more advanced cancers can be improved [20]. Since these types of treatments are more recent, the patients included in our cohort could not benefit from them.

Unfortunately, advanced stages, in particular of oral cavity and oropharynx cancers, were predominant and naturally lead to the low survival rate observed in our study. This is an important issue that must be solved in Portugal. This could be related to delays in the diagnosis of oral and oropharynx cancers [21]. A large proportion of the population does not know the early symptoms or clinical manifestations of oral cancer or are not even aware of this disease [22]. Health service interventions for head and neck cancer control should be implemented. Recently, a national programme to help early recognition of precancerous lesions and malignant tumours was introduced in Portugal [23]. An Early Detection Programme should involve researchers in order to better understand the biology of the disease, to find it earlier and to reduce the number of deaths caused by cancer. In addition, prevention strategies for the individual and society should also be developed. A cancer education programme should be implemented. These activities should be audited and epidemiological studies conducted to assess the results [24].

This study presents some limitations and strengths. Information on some variables was scarce especially on risk factors and some cases had no information on tumour stage and patient’s outcome. We used multiple imputations to deal with the missing stage information. We grouped posterior tongue (C01) within the oral cavity according to previous studies [2] and to be able to make comparisons with others. Oropharynx cancer has been associated with HPV infection with particular characteristics including better survival than HPV negative cases [25, 26]. Unfortunately, we do not have information on the HPV status of our tumours but it is likely that a part of these oropharynx tumours are related to HPV. We also adapted some novel methodologies. For survival determination, we used the net-survival method based on the Pohar-Perme estimator that has been described as the only unbiased estimator of net survival [27, 28]. For comparisons among the different variables, we also used multivariable EHRs. The strengths of the study included a population-based design with almost 3,000 patients diagnosed over a decade in the north of Portugal. Our study provides information on the survival at 5 years including the trends over this ten period time and includes an analysis by clinical stage and tumour location.

## Conclusion

The data presented here showed low net survival rates for oral cavity and oropharynx cancers in the north of Portugal in the time period of 2000–2009 with more than half of cases being diagnosed in advanced stages of the disease. Nevertheless, an apparent improvement in net survival from 2000–4 to 2005–9 was observed although limited to intermediate stages (II and III).

Thus, in order to increase survival at all stages, we need effective and concrete actions for population education, early diagnosis, better multidisciplinary treatment approaches, supportive care and a suitable follow-up.

## Funding statement

This study had no specific funding and none of the authors received any funding related to this work.

## Conflicts of interest

The authors declare that there are no potential conflicts of interest.

## Figures and Tables

**Table 1.** The description of the cohort of patients analysed.

Variable	All sites		Lip		Oral cavity		Oropharynx	
	*N*	%	*n*	%	*N*	%	*n*	%
Total	2947	100	544	100	1656	100	747	100
*Gender*								
Male	2342	79.5	358	65.8	1286	77.7	698	93.4
Female	605	20.5	186	34.2	370	22.3	49	6.6
*Age group*								
15–44	357	12.1	34	6.3	216	13.0	107	14.3
45–54	712	24.2	38	7.0	413	24.9	261	34.9
55–64	725	24.6	87	16.0	419	25.3	219	29.3
65–74	619	21.0	158	29.0	348	21.0	113	15.1
75+	534	18.1	227	41.7	260	15.7	47	6.3
Stage at diagnosis*								
I	398	18.6	195	69.6	187	14.1	16	3.0
II	302	14.1	50	17.9	200	15.1	52	9.7
III	399	18.6	18	6.4	256	19.3	125	23.3
IV	1044	48.7	17	6.1	683	51.5	344	64.1
Unknown	804		264		330		210	
Typology of residence area*								
Rural	325	11.1	108	20.0	284	17.2	128	17.2
Medium urban	520	17.7	103	19.0	162	9.8	60	8.0
Urban	2096	71.3	330	61.0	1208	73.0	558	74.8
Unknown	6		3		2		1	
*Period of diagnosis*								
2000–2004	1275	43.3	288	52.9	691	41.7	296	39.6
2005–2009	1672	56.7	256	47.1	965	58.3	451	60.4

* percentage within cases with information

**Table 2.** Net survival at 5 years of follow-up by tumour site.

Variable	5yr-NS							
	All sites		Lip		Oral cavity		Oropharynx	
	%	95%CI	%	95%CI	%	95%CI	%	95%CI
All	46	44–48	88	83–94	41	38–43	27	23–30
*Gender*								
Male	41	39–43	86	80–93	37	33–39	26	23–29
Female	64	59–69	89	80–98	54	48–60	34	20–48
*Age group*								
15–44	42	37–47	94	86–100	42	36–49	25	16–33
45–54	36	33–40	91	82–100	38	33–42	26	21–31
55–64	43	39–47	89	81–97	42	37–47	25	19–31
65–74	50	45–54	89	82–97	39	33–44	28	19–37
75+	60	53–67	85	73–96	43	34–51	35	18–53
*Stage at diagnosis*								
I	84	78–90	93	84–100	76	68–84	66	41–92
II	69	63–76	87	71–100	68	60–76	55	40–70
III	42	37–47	60	31–89	44	37–51	34	25–43
IV	19	16–21	26	0–54	20	17–23	16	12–20
*Typology of residence area*								
Predominantly rural	52	46–59	95	84–100	36	28–44	24	12–35
Medium urban	45	40–50	87	74–100	39	33–45	24	17–32
Predominantly urban	45	42–47	86	78–92	41	38–44	27	23–31
*Period of diagnosis*								
2000–2004	46	42–49	85	78–93	38	34–42	24	18–29
2005–2009	46	43–49	91	83–99	41	39–45	28	24–33

**Table 3.** Net survival at 5 years of follow-up by a period of diagnosis.

Variable	5yr-NS			
	Period 2000–2004		Period 2005–2009	
	%	95%CI	%	95%CI
All	46	42–49	46	43–49
*Gender*				
Male	41	37–44	42	38–44
Female	66	59–74	62	56–68
*Age group*				
15–44	43	35–51	41	35–48
45–54	35	29–40	37	33–42
55–64	46	40–51	41	36–46
65–74	48	41–54	52	46–58
75+	57	47–67	63	54–72
*Topography*				
Lip	85	78–93	91	83–100
Oral cavity	38	34–42	42	39–45
Oropharynx	24	19–29	28	24–33
*Stage at diagnosis*				
I	85	77–94	82	73–91
II	62	53–72	76	67–84
III	32	25–40	47	40–54
IV	18	14–22	19	16–22
*Typology of residence area*				
Predominantly urban	43	40–47	46	43–49
Medium urban	54	46–62	40	34–46
Predominantly rural	48	39–58	55	46–64

**Table 4.** Non-adjusted and adjusted EHRs.

Variable	Non-adjusted		Adjusted	
	EHR^a^	95%CI	EHR^a^	95%CI
*Gender*				
Male	1		1	
Female	0.48	0.41–0.57	0.69	0.58–0.82
*Age group*				
15–44	1		1	
45–54	1.16	0.99–1.36	0.98	0.83–1.16
55–64	1.01	0.85–1.19	1.07	0.90–1.27
65–74	0.81	0.67–0.97	1.14	0.95–1.38
75+	0.62	0.50–0.77	1.41	1.13–1.76
*Topography*				
Oral cavity			1	
Lip	0.09	0.05–0.14	0.27	0.18–0.42
Oropharynx	1.44	1.29–1.60	1.15	1.03–1.29
*Stage*				
I	1		1	
II	1.00	1.84–4.11	1.77	1.20–2.61
III	1.09	4.73–9.58	3.85	2.69–5.51
IV	4.21	10.1–19.6	7.79	5.56–10.9
*Typology of residence area*				
Predominantly urban	1		–	–
Intermediate	0.95	0.83–1.09	–	–
Predominantly rural	0.85	0.71–1.01	–	–
*Period of diagnosis*				
2000–2004			1	
2005–2009	0.99	0.89–1.09	0.82	0.74–0.91

a Each EHR is adjusted for the remaining variables.

**Table 5.** Adjusted EHRs stratified by stage at diagnosis.

Variable	Stage I		Stage II		Stage III		Stage IV	
	EHR	95%CI	EHR	95%CI	EHR	95%CI	EHR	95%CI
*Period of diagnosis*								
2000–2004	1		1		1		1	
2005–2009	1.18	0.66–2.12	0.63	0.42–0.93	0.61	0.48–0.77	0.91	0.80–1.03
